# Assessing the Impact of a Prescription Communication Skills Training Module on the Attitudes and Competency of Medical Undergraduates

**DOI:** 10.7759/cureus.82607

**Published:** 2025-04-19

**Authors:** Padmanabha Thiruganahalli Shivaraju, Ravi Shankar Manchukonda, Tejaswi H Lokanathan, Haradanahalli G Kshamaa

**Affiliations:** 1 Pharmacology, Adichunchanagiri Institute of Medical Sciences, Adichunchanagiri University, BG Nagara, IND; 2 Pharmacology, PESU Institute of Medical Sciences and Research, Bengaluru, IND; 3 Anatomy, Adichunchanagiri Institute of Medical Sciences, Adichunchanagiri University, BG Nagara, IND; 4 Psychiatry, Kempegowda Institute of Medical Sciences, Bengaluru, IND

**Keywords:** assessment, attitudes, communication module, communication skills attitude scale, competency, confidence, intervention, prescription communication skills, self-evaluation, training

## Abstract

Introduction: Good doctor-patient communication is critical in enhancing treatment adherence. Ineffective doctor communication has been associated with high patient non-adherence, while good communication improves doctor-patient relationships and improves adherence. Communication skills have been identified by the National Medical Commission as one of the important competencies for Indian medical graduates. Ineffective communication of prescriptions is still a challenge, impacting patient outcomes. Incorporating a communication skills module as per Competency-Based Medical Education (CBME) guidelines can increase the confidence and competence of medical students in communicating prescriptions to eventually improve patient care.

Methods: This interventional mixed-method study assessed the impact of prescription communication training module in 145 second-phase MBBS students after obtaining ethical clearance. The communication skills module was first introduced to the students through a sensitization session. Questionnaire on communication skills was administered to measure baseline attitudes toward communication skills. Intervention included structured training module comprised of four sessions, each of two-hour duration with role-play. Upon completion of the sessions, a post-test utilizing the same questionnaire on attitudes was administered to measure differences in communication skills attitudes. The main outcome was assessed by change in scores of communication skills before and after the module training using objective structured practical examination (OSPE). Students' reactions towards the training module were recorded. Data were analyzed for statistical significance using t-test.

Results: The post-test was taken by 124 (85.52% response rate) out of 145 medical students in second phase. Positive attitude scores in the post-intervention phase were more favorable in male students, rural-background students, and doctor-families, whereas negative attitude scores were negatively affected in males and urban-background students. Student performance above 6.5 scores improved from 47.58% (n = 59) to 57.26% (n = 71) after intervention, which was statistically significant (p value < 0.001). Ability to communicate rated as "very good" improved to 9.68% (n =12) from 5.34% (n =7). 87% (n = 34) of the males and 100% (n = 86) of the females concurred that training done regularly would enhance their performance.

Conclusion:Students' performance and self-confidence in prescription communication skills improved as a result of the four-week training program. However, there was no appreciable shift in attitudes. Inclusion of such modules in healthcare education would make prescription communication competent and confident for students. Additional studies are needed to determine its actual effect in real-life practice on patient communication.

## Introduction

Adherence of the patient to the treatment has been attributed to the doctor-patient communication. Meta-analysis has revealed that the effective communication can increase the adherence of the patients by 19% [1]. The likelihood of patient adherence increased by 2.16 times when doctors communicated effectively with their patients. [1]. The National Medical Commission (formerly Medical Council of India) has made communication skills one of the seven pre-defined competencies to be attained by an Indian medical graduate under medical education [2]. To rectify defective prescription communication, inclusion of an exhaustive module on communication skills as per Competency-Based Medical Education (CBME) guidelines would improve prescription and communication skills of MBBS students and maximize outcomes in patients [2]. The attitudes and confidence of medical students in this area are not well understood, despite the fact that prescription communication is crucial for patient outcomes. This study aims to bridge that gap. Therefore, the objectives of this research were to quantify positive and negative attitudes towards prescription communication skills among medical students, establish the impact of a prescription communication skills training module, and compare their perception towards prescription communication skills training.

## Materials and methods

Study setting

In this interventional mixed-method study, both quantitative and qualitative approaches were integrated in the Department of Pharmacology, Adichunchanagiri Institute of Medical Sciences, from July 2022 to December 2022.

Study population and sampling

Second-phase MBBS students (n = 145) were recruited for prescription communication module by convenience sampling.

Ethics approval

Ethical clearance was taken from Institutional Ethics Committee of Adichunchanagiri Institute of Medical Sciences (approval number AIMS/IEC/020/2022, dated June 11, 2022). Written informed consent was taken from the students after orientation on prescription communication skills sessions.

Study instruments

**Figure 1:**
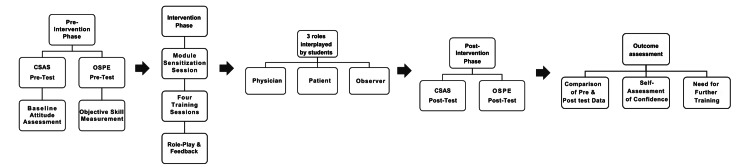
Process of structured prescription communication skills module implementation CSAS: Communication skills attitude scale; OSPE: Objective structured practical examination

Communication skills attitude scale (CSAS) was used to know the students perception towards communication [3]. It includes 26 items with Positive Attitude Subscale (PAS) having 13 items and Negative Attitude Subscale (NAS) having 13 items. Each item is scored from 1 (strongly disagree) to 5 (strongly agree), with higher scores indicating stronger attitudes. CSAS was administered before and after the intervention using Google Form, and 15 minutes were provided to respond to CSAS. Kalamazoo Essential Elements Communication Checklist (Adapted) (KEECC-A) has also been validated as a reliable tool for assessing communication skills [4]. It includes seven essential elements for prescription communication in clinical situations: build the relationship, open the discussion, gather information, understand the patient's perspective, share information, reach agreement, and provide closure. Case scenarios for diabetes mellitus, hypertension, migraine, epilepsy and bronchial asthma were prepared based on the elements of KEECC-A. Structured scripts for the facilitator and students included concise information on patient and physician briefing hand-outs during four sessions. Clinicians and the pharmacology department peer-reviewed the training modules and suggested changes that were implemented. Facilitators were trained to give constructive feedback to all students on training session performance. OSPE was conducted using KEECC-A before and after the training module. During OSPE, students were given 8 to 10 minutes to communicate the prescription-related information to the facilitator who acted as patient as well as assessor. OSPE scores obtained before and after were used to assess the impact of training sessions (Figure 1).

Methodology

Pre-intervention phase included CSAS pre-test and OSPE (Figure 1). Pre-test was given to evaluate students' initial attitude towards prescription communication competence using CSAS in relation to demographic characteristics (gender, location, education, presence of doctors in the family, and fluency in the local language). OSPE was used to evaluate objectively students' prescription communication skills prior to intervention. It was carried out by case-based scenarios of the training module where the students were requested to communicate a single item of prescription information to simulated patients and facilitator as observer for evaluation using. A KEECC-A checklist with each item was scored on 1 to 10 scale and total average score of 10 was calculated. Scores below 4, 4-6.5, and above 6.5 were graded as below average, average, and above average performers, respectively. Minimum eligibility criteria for a student to get eligible for final examination as per CBME curriculum was 40%: this was the basis to categorize the students into three performance groups [2].

The intervention phase included sensitization session on prescription communication skills training module and four training sessions in the students' regular pharmacology practical classes (Figure 1). Students were initially briefed about communication skill components (verbal and non-verbal) and medication counseling technical skill (problem-solving skill). Every session began with a clinical case scenario briefing by the facilitator, then a small group discussion by students took place, supplemented with video presentation on communication, and the session concluded with role-play by three students. Students were allowed to opt for either physician or patient or observer roles and switch roles during subsequent sessions. Students playing the patient and physician possessed communication role scripts, and observer carried a checklist for evaluation and provision of structured feedback. The facilitator conducting the session provided constructive feedback on communication issues for targeted case scenarios.

Post-intervention phase at the end of four training sessions included CSAS post-test to measure attitude change in prescription communication and post-intervention OSPE used the same case-based situations of the training module to measure improvement in communication skills of students.

Outcome evaluation

Pre-intervention and post-intervention data of students who attended all four sessions were compared to determine the efficacy of the training module. Apart from that, they were also requested to indicate the level of confidence they have in prescription communication skills both before and after the training session on a 5-point Likert scale from "very good" (5 marks) to "very poor" (1 mark) and requested their feedback for the necessity of periodic training to facilitate them in developing their communication skills (Figure 1). Kirkpatrick’s four-level evaluation model was used to evaluate the training module [5].

Statistical analysis

Information collected were entered to Microsoft Excel data sheet and the statistical analysis performed utilizing IBM SPSS Statistics for Windows, Version 26.0 (IBM Corp., Armonk, USA). Categorical data were reported in frequency and proportion. Pre-intervention and post-intervention mean scores were compared with paired t-test (two sample unequal and equal variance). P < 0.05 was set for statistical significance. Mean and SD were utilized in descriptive analysis.

## Results

Of the 145 students, 131 (90.34% response rate) completed the pre-intervention, while 124 (85.52% response rate) completed the post-intervention.

The CSAS scale demonstrated high internal consistency, with Cronbach's alpha being 0.926 in the pre-intervention and 0.915 in the post-intervention.

The mean NAS scores in PAS dropped slightly from 54.22 to 53.81, whereas NAS increased marginally to 34.98 from 34.73. The percentage change for attitude was marginal, and none of the differences were statistically significant (Table 1).

**Table 1:** CSAS: PAS and NAS mean scores comparison between pre- and post-intervention phase CSAS: Communication Skills Attitude Scale; PAS: Positive Attitude Subscale; NAS: Negative Attitude Subscale *p-value less than 0.05 considered statistically significant (t-test: two-sample unequal variance)

CSAS	Pre-intervention phase (n = 131)		Post-intervention phase (n = 124)		Percentage (%) change in the attitude	p-value*
	Mean	SD	Mean	SD		
PAS	54.22	6.9	53.81	8.07	-0.01	0.41
NAS	34.73	6.3	34.98	8.83	0.01	0.91

Post-intervention CSAS mean scores for PAS revealed that positive attitude among urban students (n = 86) improved marginally (53.20 to 53.71), whereas rural students (n = 38) marginally decreased (56.20 to 54.68). Male students marginally improved (52.10 to 52.94), whereas female students' scores decreased (55.00 to 54.04). Students with a doctor in the family marginally improved (54.00 to 54.58), whereas students without a doctor in the family marginally decreased (54.70 to 53.51). Kannada-speaking students registered a slight decline (54.50 to 53.82), and the non-Kannada-speaking students also registered a slight decline (53.10 to 52.82). The other groups remained unchanged or showed negligible change, and all p-values were not significant (Table 2).

**Table 2:** Comparison of PAS mean scores and its demographic variables between pre- and post-intervention phase PAS: Positive Attitude Subscale; ICSE: Indian Certificate of Secondary Education; CBSE: Central Board of Secondary Education *p-value less than 0.05 considered statistically significant (t-test: two-sample unequal variance)

Variables		PAS mean scores						Percentage (%) change in the attitude	p-value*
		Pre-intervention phase (n = 131)			Post-intervention phase (n = 124)				
		n	Mean	SD	n	Mean	SD		
Gender	Male	37	52.10	7.40	39	52.94	7.50	0.02	0.97
	Female	94	55.00	6.60	85	54.04	8.27	-0.02	
Location	Rural	44	56.20	6.10	38	54.68	11.50	-0.03	0.79
	Urban	87	53.20	9.80	86	53.71	7.19	0.01	
Syllabus	State syllabus	56	54.50	9.10	59	53.82	11.30	-0.01	0.47
	ICSE/CBSE	75	53.90	7.10	65	53.93	6.37	0.00	
Family background	Doctors in family	94	54.00	6.60	41	54.58	7.83	0.01	0.68
	No doctors in family	37	54.70	7.80	83	53.51	8.25	-0.02	
Language	Kannada speaking	108	54.40	6.90	101	53.89	8.40	-0.01	0.68
	Non-Kannada speaking	23	53.10	6.60	23	52.82	6.19	-0.01	

The NAS mean scores varied with some demographic factors before and after the intervention. Decrease in scores indicates reduced negative attitude, and increase indicates an increase. Rural students decreased negative attitude (36.50 to 34.55), and urban students experienced a small increase (33.80 to 35.17). Non-Kannada-speaking students experienced an increase in negative attitude (35.40 to 37.08). State syllabus students experienced a small increase (35.75 to 36.06). Both with and without a doctor in the family showed a marginal rise in negative attitude (34.70 to 34.95 and 34.80 to 35.00, respectively). The remaining groups, i.e., male, and female students, Indian Certificate of Secondary Education (ICSE)/Central Board of Secondary Education (CBSE) students, and Kannada-speaking students, showed no change to negligible change. All p-values were not significant (Table 3).

**Table 3:** Comparison of NAS mean scores and its demographic variables between pre- and post-intervention phase NAS: Negative Attitude Subscale; ICSE: Indian Certificate of Secondary Education; CBSE: Central Board of Secondary Education *p-value less than 0.05 considered statistically significant (t-test: two-sample unequal variance)

Variables		NAS mean scores						Percentage (%) change in the attitude	p-value*
		Pre-intervention phase (n = 131)			Post-intervention phase (n = 124)				
		n	Mean	SD	n	Mean	SD		
Gender	Male	37	37.20	6.60	39	37.02	9.27	0.00	0.99
	Female	94	33.80	5.90	85	34.04	8.52	0.01	
Location	Rural	44	36.50	6.00	38	34.55	8.86	-0.05	0.86
	Urban	87	33.80	12.10	86	35.17	8.86	0.04	
Syllabus	State syllabus	56	35.75	7.10	59	36.06	9.86	0.01	0.89
	ICSE/CBSE	75	33.90	5.40	65	34.00	7.73	0.00	
Family background	Doctors in family	94	34.70	6.10	41	34.95	7.73	0.01	0.09
	No doctors in family	37	34.80	6.18	83	35.00	9.37	0.01	
Language	Kannada speaking	108	34.50	6.30	101	34.50	8.44	0.00	0.63
	Non-Kannada speaking	23	35.40	6.30	23	37.08	10.31	0.05	

"Very good" confidence level increased considerably post intervention (81%). "Good" and "Satisfactory" levels of confidence dropped (-2% and -11%, respectively). "Very poor" and "Poor" ratings remained unchanged with a minimal 6% rise and the changes observed was not statistically significant (Table 4).

**Table 4:** Comparison of self-rating of student's confidence on practicing prescription communication skills *p-value less than 0.05 considered statistically significant (t-test: two-sample equal variance)

Likert Scale	Pre-intervention phase		Post-intervention phase		Percentage (%) change in perception	p-value*
	Frequency (n = 124)	Percentage (%)	Frequency (n = 124)	Percentage (%)		
Very good	7	5.34	12	9.68	81	> 0.05
Good	84	64.12	78	62.90	-2	
Satisfactory	38	29.01	32	25.81	-11	
Poor	1	0.76	1	0.81	6	
Very poor	1	0.76	1	0.81	6	

**Figure 2:**
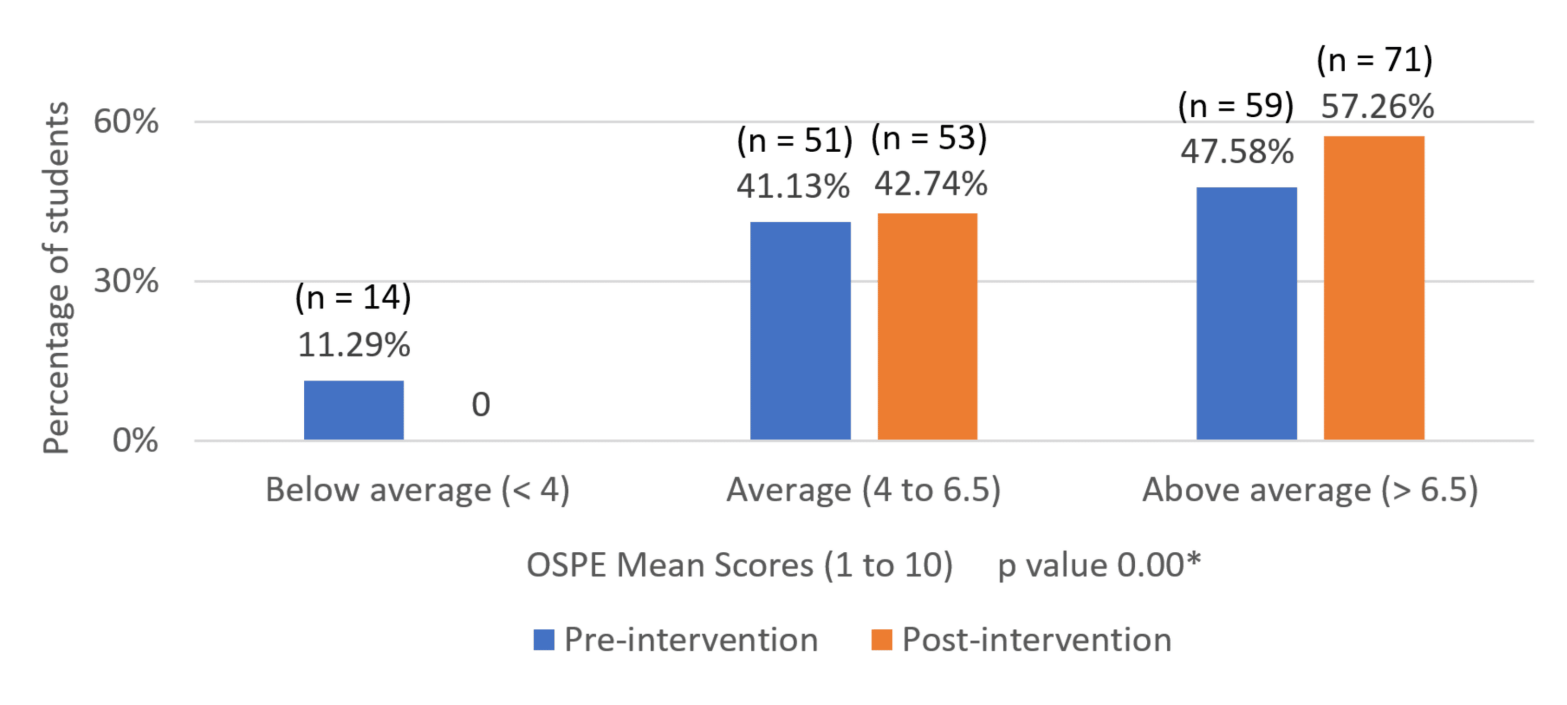
Comparison of OSPE mean scores for prescription communication skills in pre- and post-intervention phase OSPE: Objective structured practical examination *p value less than 0.001 considered statistically significant (t-test: two-sample equal variance)

Following the intervention, the percentage of students who had a learning level of less than 4 dropped from 11.29% (n = 14) to 0%. The proportion of students in the 4-6.5 range increased slightly from 41.13% (n = 51) to 42.74% (n = 53). Also, the percentage of students who had a learning level of more than 6.5 went up from 47.58% (n = 59) to 57.26% (n = 71) (Figure 2).

**Figure 3:**
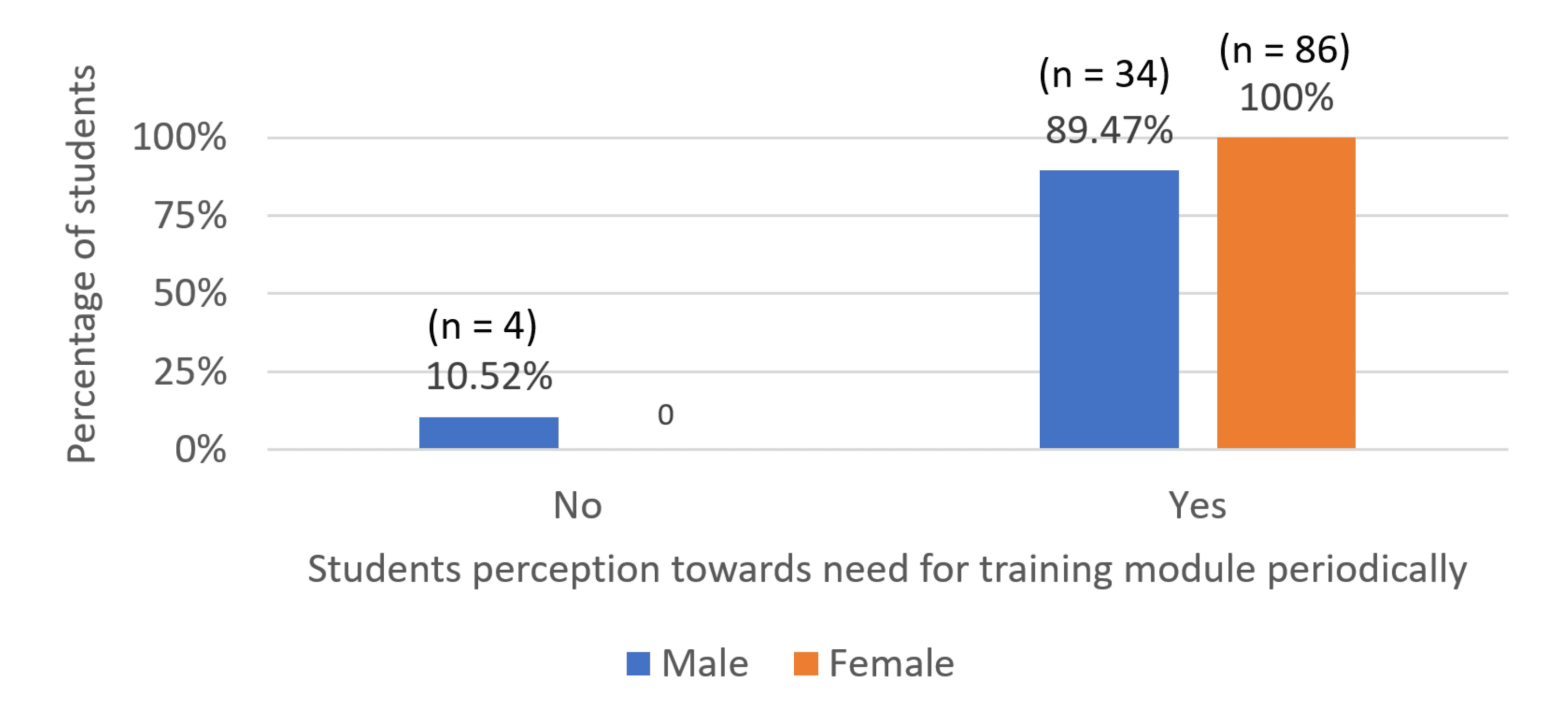
Students perception on need for further training to improve their skills following intervention

Of the male students, 89.47% (n = 34) and 100% (n = 86) of the female students stated that they need to be trained on a regular basis to strengthen their skills (Figure 3).

## Discussion

Our research found few differences in CSAS scores after the intervention, with no statistically significant differences. PAS scores decreased slightly (54.22 to 53.81), whereas NAS scores increased slightly (34.73 to 34.98) (Table 1). This is the opposite of what was revealed in studies by Tanrıverdi et al. and Cleland et al., which showed dramatic improvement in PAS and deterioration in NAS following formal training in communication skills [6,7]. The reason could be due to limited exposure to the communication skills training module that could have prevented it from being as effective as compared to studies with longer, repeated, or immersive training.

Male students posted a mild increase in PAS (52.10 to 52.94), while females posted a decline (55.00 to 54.04) (Table 2). This is not in line with the earlier research conducted by Busch et al. [8], Ezeala et al. [9] who reported higher PAS scores and lower NAS scores in female students [8,9]. In our study, negative attitude decreased in rural students (36.50 to 34.55), following the intervention, however it increased in urban students (33.80 to 35.17) (Table 3). After the intervention, rural students exhibited greater positive attitude and reduced negative attitude than urban students, consistent with our previous work on attitude towards CSAS need assessment [10]. Students who spoke other languages were seen to have higher scores on negative attitude on NAS (35.40 to 37.08) following intervention. Although no clear reason has been found, it may be secondary to discomfort in communicating in the local language, supporting the evidence that attitudes towards communication skills are influenced by language [4]. These differences in the populations can be attributed to observed differences in attitude change, perhaps due to variation in exposure level, cultural environment, or comfort with communication skills training between populations.

Despite these small changes in attitudes, our research showed enhancement in students' communication performance and self-reported confidence following the intervention. Overall scores improved from an average of 6.14 pre-test to 7.27 after the intervention. The below-average group (<4) dropped to 0%, and the above-average group (>6.5) increased from 47.58% (n = 59) to 57.26% (n = 71), and the same was statistically significant (p 0.00) (Figure 2). The confidence self-perception also increased by 81% in the "Very good" category but not statistically significant. This is consistent with previous studies using the KEECC-A for training modules [11-13]. The enhanced communication performance and confidence may have been a result of competency-based training and application of KEECC-A, most probably had a significant effect by offering definite assessment criteria, enabling students to pinpoint strengths and weaknesses in communication abilities.

Moreover, 89.47% (n = 34) of male students and 100% (n = 86) of female students recognized the need for further improvement even after the intervention (Figure 3). This suggests that while the training was beneficial, students were still aware of their ongoing need for learning, emphasizing the importance of regular training. However, as Ruiz-Moral et al. have noticed, these positive changes in attitude towards communication skills was lost after some time in the absence of ongoing follow-up, emphasizing the importance of regular training and monitoring [14]. The increase in understanding in the students regarding the need for improvement of prescription communication skills suggests that the training enhanced the student self-perception of their communication ability, validating that skill competence requires constant learning and refreshing.

Evidence indicates that a systematic experiential strategy might be more effective in inducing attitudinal change, as is illustrated in work by Ezeala et al. and Akbarilakeh et al. [9,15]. In addition, literature emphasizes the necessity of systematic training and feedback in communicating skill development [11,12]. Variation in improvement, though, implies greater standardization is required [16].

Finally, our study reached three levels of Kirkpatrick's four-level evaluation model [5]. Level 1 (Reaction) was reached as the students were confident with their communication skill in their response. Level 2 (Learning) was reached by assessing improvement in students' performance through OSPE, and a minor shift in attitude. Level 3 (Behavior) was attained by enhancing students motivation to use such training modules on a routine basis to improve their capabilities.

Limitations to the study were its short period of conduct, small sample, single institution, one-student phase, potential self-report bias, and absence of follow-up. Varying background and training experience of participants potentially influenced findings and, hence, the importance of guided interventions of long duration.

## Conclusions

The four-week module included KEECC-A-based training improved performance, self-confidence, and communication competence among students. However, minimal attitudinal changes were acknowledged, demonstrating that a more systematic, longitudinal approach is needed to achieve a lasting impact in real-life practice. Implementation of training module at regular intervals in the curriculum may result in improved communication skills in the students.
